# New Type Continuities via Abel Convergence

**DOI:** 10.1155/2014/398379

**Published:** 2014-04-27

**Authors:** Huseyin Cakalli, Mehmet Albayrak

**Affiliations:** ^1^Department of Mathematics, Maltepe University, Marmara Eğıtım Köyü, Maltepe, 34857 İstanbul, Turkey; ^2^Department of Mathematics, Sakarya University, Sakarya, 54050 İstanbul, Turkey

## Abstract

We investigate the concept of Abel continuity. A function *f* defined on a subset of ℝ, the set of real numbers, is Abel continuous if it preserves Abel convergent sequences. Some other types of continuities are also studied and interesting result is obtained. It turned out that uniform limit of a sequence of Abel continuous functions is Abel continuous and the set of Abel continuous functions is a closed subset of continuous functions.

## 1. Introduction

The concept of continuity and any concept involving continuity play a very important role not only in pure mathematics but also in other branches of sciences involving mathematics especially in computer sciences, information theory, and dynamical systems.

A method of sequential convergence is a linear function *G* defined on a linear subspace of *s*, denoted by *c*_*G*_, into *R* where *R* and *s* denote the set of real numbers and the space of all sequences, respectively. A sequence **p** = (*p*_*n*_) is said to be *G*-convergent to *l* if **p** ∈ *c*_*G*_, and *G*(**p**) = *l* [1]. A method *G* is called regular if every convergent sequence **p** = (*p*_*n*_) is *G*-convergent with *G*(**p**) = lim⁡⁡**p**. A method *G* is called subsequential if whenever **p** is *G*-convergent with *G*(**p**) = *l*, then there is a subsequence (*p*_*n*_*k*__) of **p** with lim⁡_*k*_ *p*_*n*_*k*__ = *l*. A function *f* is called *G*-continuous (see also [2, 3]) if *G*(*f*(**p**)) = *f*(*G*(**p**)) for any *G*-convergent sequence **p**. Any matrix summability method on a subspace of *s* is a method of sequential convergence. Abel summability method is a regular method of sequential convergence in this manner.

The purpose of this paper is to investigate the concept of Abel continuity for real functions and present interesting results.

## 2. Definitions and Notations and Preliminary Results

We will use boldface letters **p**,**r**,**w**,…, for sequences **p** = (*p*_*n*_), **r** = (*r*_*n*_), **w** = (*w*_*n*_),… of points in *R*. A sequence **p** = (*p*_*k*_) of points in *R* is called statistically convergent [4] (see also [5–9]) to an element *l* of *R* if

(1) $$\begin{array}{c} \underset{n\to \infty }{\lim}\frac{1}{n}\left|\left\{k\leq n:\left|p_{k}-l\right|\geq ɛ\right\}\right|=0, \end{array}$$

for every *ɛ* > 0, and this is denoted by st-lim⁡*p*_*k*_ = *l*.

A sequence (*p*_*k*_) of points in *R* is called lacunary statistically convergent [10] to an element *l* of *R* if

(2) $$\begin{array}{c} \underset{r\to \infty }{\lim}\frac{1}{h_{r}}\left|\left\{k\in I_{r}:\left|p_{k}-l\right|\geq ɛ\right\}\right|=0, \end{array}$$

for every *ɛ* > 0, where *I*_*r*_ = (*k*_*r*−1_, *k*_*r*_], *k*_0_ = 0, *h*_*r*_ : *k*_*r*_ − *k*_*r*−1_ → *∞* as *r* → *∞*, and *θ* = (*k*_*r*_) is an increasing sequence of positive integers, and this is denoted by *S*_*θ*_-lim⁡*p*_*n*_ = *l* (see also [11–13]). Throughout this paper we assume that lim⁡ inf⁡_*r*_(*k*_*r*_/*k*_*r*−1_) > 1. A sequence **p** = (*p*_*n*_) of points in *R* is slowly oscillating [14] (see also [15, 16]), denoted by **p** ∈ **S** **O**, if $\lim_{\lambda \to 1^{+}}{\overset{-}{\lim}}_{n}\max_{n+1\leq k\leq [\lambda n]}\left|x_{k}-x_{n}\right|$ where [*λn*] denotes the integer part of *λn*.

A sequence **p** = (*p*_*n*_) of real numbers is called Abel convergent (or Abel summable) to *l* if the series Σ_*k*=0_^*∞*^ *p*_*k*_ *x* ^*k*^ is convergent for 0 ≤ *x* < 1 and

(3) $$\begin{array}{c} \underset{x\to 1^{-}}{\lim}\left(1-x\right)\overset{\infty }{\underset{k=0}{\sum }}p_{k}x^{k}=l. \end{array}$$

In this case we write Abel-lim⁡*p*_*n*_ = *l*. The set of Abel convergent sequences will be denoted by **A**. Abel proved that if lim⁡_*n*→*∞*_ *p*_*n*_ = *l*, then Abel-lim⁡*p*_*n*_ = *l*; Abel sequential method is regular; that is, every convergent sequence is Abel convergent to the same limit ([17]; see also [18, 19]). As it is known that the converse is not always true in general, we see that the sequence ((−1)^*n*^) is Abel convergent to 0 but convergent in the ordinary sense.

**Definition 1**

A subset *E* of *R* is called Abel sequentially compact if, whenever **p** = (*p*_*n*_) is a sequence of points in *E*, there is an Abel convergent subsequence **r** = (*r*_*k*_) = (*r*_*n*_*k*__) of **p** with lim⁡_*x*→1^−^_(1 − *x*)∑_*k*=0_^*∞*^ *r*_*k*_ *x* ^*k*^ ∈ *E*.

**Definition 2**

A real number *l* is said to be in the Abel sequentially closure of a subset *E* of *R*, denoted by ${\bar{E}}^{\text{Abel}}$, if there is a sequence **p** = (*p*_*n*_) of points in *E* such that Abel-lim⁡*p*_*n*_ = *l*, and it is called Abel sequentially closed if ${\bar{E}}^{\text{Abel}}=E$.

Note that the preceding definitions are special cases of the definitions of *G*-sequential compactness and *G*-sequential closure in [2].

It is clear that ${\bar{\phi }}^{\text{Abel}}=\phi$ and ${\bar{R}}^{\text{Abel}}=R$. It is easily seen that $E\subset \bar{E}\subset {\bar{E}}^{\text{Abel}}$. It is not always true that ${\bar{({\bar{E}}^{\text{Abel}})\;}}^{\text{Abel}}={\bar{E}}^{\text{Abel}}$; for example, ${\bar{\left({\bar{\left\{-1,1\right\}}}^{\text{Abel}}\right)}}^{\text{Abel}}\neq {\bar{\left\{-1,1\right\}}}^{\text{Abel}}$. We note that any Abel sequentially closed subset of Abel sequentially compact subset of *R* is also Abel sequentially compact. Thus intersection of two Abel sequentially compacts, Abel sequentially closed subsets of *R*, is Abel sequentially compact. In general, any intersection of Abel sequentially compact and Abel sequentially closed subsets of *R* is Abel sequentially compact. The condition closedness is essential here; that is, a subset of an Abel sequentially compact subset need not to be Abel sequentially compact. For example, the interval ] − 1,1], that is, the set of real numbers strictly greater than −1 and less than or equal to 1, is a subset of Abel sequentially compact subset [−1,1], that is, the set of real numbers greater than or equal to −1 and less than or equal to 1, but not Abel sequentially compact. Notice that union of two Abel sequentially compact subsets of *R* is Abel sequentially compact. We see that any finite union of Abel sequentially compact subsets of *R* is Abel sequentially compact, but any union of Abel sequentially compact subsets of *R* is not always Abel sequentially compact.

## 3. Main Results

First we introduce two notions.

**Definition 3**

A sequence (*p*_*k*_) of point in *R* is called Abel quasi-Cauchy if (Δ*p*_*k*_) is Abel convergent to 0; that is,

(4) $$\begin{array}{c} \underset{x\to 1^{-}}{\lim}\left(1-x\right)\overset{\infty }{\underset{k=0}{\sum }}\Delta p_{k}x^{k}=0, \end{array}$$

where Δ*p*_*k*_ = *p*_*k*+1_ − *p*_*k*_ for each positive integer *k*.

**Definition 4**

A subset *E* of *R* is called Abel ward compact if, whenever **p** = (*p*_*n*_) is a sequence of point in *E* there is an Abel quasi-Cauchy subsequence **r** = (*r*_*k*_) = (*r*_*n*_*k*__) of **p**; that is, lim⁡_*x*→1^−^_(1 − *x*)∑_*k*=0_^*∞*^Δ*r*_*k*_ *x* ^*k*^ = 0.

We note that any Abel sequentially compact subset of *R* is also Abel ward compact. Intersection of two Abel ward compact subsets of *R* is Abel ward compact. In general, any intersection of Abel ward compact subsets of *R* is Abel ward compact. Notice that union of two Abel ward compact subsets of *R* is Abel ward compact. We see that any finite union of Abel sequentially compact subsets of *R* is Abel ward compact, but any union of Abel ward compact subsets of *R* is not always Abel ward compact.

**Theorem 5**

A subset *A* of *R* is bounded if and only if it is Abel ward compact.

**Proof**

It is an easy exercise to check that bounded subsets of *R* are Abel ward compact. To prove that Abel ward compactness implies boundedness, suppose that *A* is unbounded. If it is unbounded above, then one can construct a sequence (*α*_*n*_) of numbers in *A* such that *α*_*n*+1_ > 2^*n*^ + *α*_*n*_ for each positive integer *n*. Then the sequence (*α*_*n*_) does not have any Abel quasi-Cauchy subsequence, so *A* is not Abel ward compact. If *A* is bounded above and unbounded below, then similarly we obtain that *A* is not Abel ward compact. This completes the proof.

We now introduce a new type of continuity defined via Abel convergent sequences.

**Definition 6**

A function *f* is called Abel continuous, denoted by **f** ∈**A** **C**, if it transforms Abel convergent sequences to Abel convergent sequences; that is, (*f*(*p*_*n*_)) is Abel convergent to *f*(*l*) whenever (*p*_*n*_) is Abel convergent to *l*.

We note that the sum of two Abel continuous functions is Abel continuous, and composite of two Abel continuous functions is Abel continuous, but the product of two Abel continuous functions need not be Abel continuous as it can be seen by considering product of the Abel continuous function *f*(*t*) = *t* with itself. We see that *G* defined by *G*(**p**) = Abel-lim⁡ **p** is a sequential method in the manner of [2], but not subsequential, so the theorems involving subsequentiality in [2] cannot be applied to Abel sequential method. In connection with Abel convergent sequences and convergent sequences the problem arises to investigate the following types of continuity of functions on *R*:

(5) $$\begin{array}{c} \left(A\right)\text{:}\;\left(p_{n}\right)\in A⟹\left(f\left(p_{n}\right)\right)\in A,\\ \left(Ac\right)\text{:}\;\left(p_{n}\right)\in A⟹\left(f\left(p_{n}\right)\right)\in c,\\ \left(c\right)\text{:}\;\left(p_{n}\right)\in c⟹\left(f\left(p_{n}\right)\right)\in c,\\ \left(cA\right)\text{:}\;\left(p_{n}\right)\in c⟹\left(f\left(p_{n}\right)\right)\in A. \end{array}$$

We see that *A* is Abel continuity of *f*, and (*c*) states the ordinary continuity of *f*. We easily see that (*c*) implies (*cA*), (*A*) implies (*cA*), and (*Ac*) implies (*A*). The converses are not always true as the identity function could be taken as a counter example for all the cases.

We note that (*c*) can be replaced by either statistical continuity; that is, st-lim⁡*f*(*p*_*n*_) = *f*(*l*) whenever **p** = (*p*_*n*_) is a statistically convergent sequence with st-lim⁡*p*_*n*_ = *l*, or lacunary statistical continuity; that is, *S*_*θ*_-lim⁡*f*(*p*_*n*_) = *f*(*l*) whenever **p** = (*p*_*n*_) is a lacunary statistically convergent sequence with *S*_*θ*_-lim⁡*p*_*n*_ = *l*. More generally (*c*) can be replaced by *G*-sequential continuity of *f* for any regular subsequential method *G*.

Now we give the implication that (*A*) implies (*c*); that is, any Abel continuous function is continuous in the ordinary sense.

**Theorem 7**

If a function *f* is Abel continuous on a subset *E* of *R*, then it is continuous on *E* in the ordinary sense.

**Proof**

Suppose that a function *f* is not continuous on *E*. Then there exists a sequence (*p*_*n*_) with lim⁡_*n*→*∞*_ *p*_*n*_ = *l* such that (*f*(*p*_*n*_)) is not convergent to *f*(*l*). If (*f*(*p*_*n*_)) exists and lim⁡*f*(*p*_*n*_) is different from *f*(*l*), then we easily see a contradiction. Now suppose that (*f*(*p*_*n*_)) has two subsequences of *f*(*p*_*n*_) such that lim⁡_*m*→*∞*_ *f*(*p*_*k*_*m*__) = *L*_1_ and lim⁡_*k*→*∞*_ *f*(*p*_*n*_*k*__) = *L*_2_. Since (*p*_*n*_*k*__) is subsequence of (*p*_*n*_), by hypothesis, lim⁡_*k*→*∞*_ *f*(*p*_*n*_*k*__) = *f*(*l*) and (*p*_*k*_*m*__) is a subsequence of (*p*_*n*_), by hypothesis lim⁡_*m*→*∞*_ *f*(*p*_*k*_*m*__) = *f*(*l*). This is a contradiction. If (*f*(*p*_*n*_)) is unbounded above, then we can find an *n*_1_ such that *f*(*p*_*n*_1__) > 2^1^. There exists a positive integer an *n*_2_ > *n*_1_ such that *f*(*p*_*n*_2__) > 2^2^. Suppose that we have chosen an *n*_*k*−1_ > *n*_*k*−2_ such that *f*(*p*_*n*_*k*−1__) > 2^*k*−1^. Then we can choose an *n*_*k*_ > *n*_*k*−1_ such that *f*(*p*_*n*_*k*__) > 2^*k*^. Inductively we can construct a subsequence (*f*(*p*_*n*_*k*__)) of (*f*(*p*_*n*_)) such that *f*(*p*_*n*_*k*__) > 2^*k*^. Since the sequence (*p*_*n*_*k*__) is a subsequence of (*p*_*n*_), the subsequence (*p*_*n*_*k*__) is convergent and so is Abel convergent. But (*f*(*p*_*n*_*k*__)) is not Abel convergent as we see in the line below. For each positive integer *k* we have *f*(*p*_*n*_*k*__)*x* ^*k*^ > 2^*k*^ *x* ^*k*^. The series ∑_*k*=0_^*∞*^2^*k*^ *x* ^*k*^ is divergent for any *x* satisfying 1/2 < *x* < 1 and so is the series ∑_*k*=0_^*∞*^ *f*(*p*_*n*_*k*__)*x* ^*k*^. This is a contradiction to the Abel convergence of the sequence (*f*(*p*_*n*_*k*__)). If (*f*(*p*_*n*_)) is unbounded below, similarly ∑_*k*=0_^*∞*^ *f*(*p*_*k*_)*x* ^*k*^ is found to be divergent. The contradiction for all possible cases to the Abel continuity of *f* completes the proof of the theorem.

The converse is not always true for the bounded function *f*(*t*) = 1/(1 + *t* ^2^) defined on *R* as an example. The function *t* ^2^ is another example which is unbounded on *R* as well.

On the other hand not all uniformly continuous functions are Abel continuous. For example, the function defined by *f*(*t*) = *t* ^3^ is uniformly continuous but Abel continuous.

**Corollary 8**

If f is Abel continuous, then it is statistically continuous.

**Proof**

The proof follows from Theorem 7, Corollary 4 in [6], Lemma 1, and Theorem 8 in [3].

**Corollary 9**

If f is Abel continuous, then it is lacunarily statistically sequentially continuous.

**Proof**

The proof follows from Theorem 7 (see [20]).

Now we have the following result.

**Corollary 10**

If (*p*_*n*_) is slowly oscillating, Abel convergent, and *f* is an Abel continuous function, then (*f*(*p*_*n*_)) is a convergent sequence.

**Proof**

If (*p*_*n*_) is slowly oscillating and Abel convergent, then (*p*_*n*_) is convergent by the generalized Littlewood Tauberian theorem for the Abel summability method. By Theorem 7, *f* is continuous, so (*f*(*p*_*n*_)) is convergent, This completes the proof.

**Corollary 11**

For any regular subsequential method *G*, any Abel continuous function is *G*-continuous.

**Proof**

Let *f* be an Abel continuous function and *G* be a regular subsequential method. By Theorem 7, the function *f* is continuous. Combining Lemma 1 and Corollary 9 of [3] we obtain that *f* is *G*-continuous.

For bounded functions we have the following result.

**Theorem 12**

Any bounded Abel continuous function is Cesaro continuous.

**Proof**

Let *f* be a bounded Abel continuous function. Now we are going to obtain that *f* is Cesaro continuous. To do this take any Cesaro convergent sequence (*p*_*n*_) with Cesaro limit *l*. Since any Cesaro convergent sequence is Abel convergent to the same value [21] (see also [22]), (*p*_*n*_) is also Abel convergent to *l*. By the assumption that *f* is Abel continuous, (*f*(*p*_*n*_)) is Abel convergent to *f*(*l*). By the boundedness of *f*, (*f*(*p*_*n*_)) is bounded. By Corollary to Karamata's Hauptsatz on page 108 in [18], (*f*(*p*_*n*_)) is Cesaro convergent to *f*(*l*). Thus *f* is Cesaro continuous at the point *l*. Hence *f* is Cesaro continuous at any point in the domain.

**Corollary 13**

Any bounded Abel continuous function is uniformly continuous.

**Proof**

The proof follows from the preceding theorem, and the theorem on page 73 in [23].

It is well known that uniform limit of a sequence of continuous functions is continuous. This is also true for Abel continuity; that is, uniform limit of a sequence of Abel continuous functions is Abel continuous.

**Theorem**

If (*f*_*n*_) is a sequence of Abel continuous functions defined on a subset E of *R* and (*f*_*n*_) is uniformly convergent to a function *f*, then *f* is Abel continuous on *E*.

**Proof**

Let (*p*_*n*_) be an Abel convergent sequence of real numbers in *E*. Write Abel-lim*p*_*n*_ = *l*. Take any *ɛ* > 0. Since (*f*_*n*_) is uniformly convergent to *f*, there exists a positive integer *N* such that

(6) $$\begin{array}{c} \left|f_{n}\left(t\right)-f\left(t\right)\right|<\frac{ɛ}{3} \end{array}$$

for all *t* ∈ *E* whenever *n* ≥ *N*. Hence

(7) $$\begin{array}{c} \left|\left(1-x\right)\overset{\infty }{\underset{k=0}{\sum }}\left(f\left(p_{k}\right)-f_{N}\left(p_{k}\right)\right)x^{k}\right|<\frac{ɛ}{3}. \end{array}$$

As *f*_*N*_ is Abel continuous, then there exist a *δ* > 0 for 1 − *δ* < *x* < 1 such that

(8) $$\begin{array}{c} \left|\left(1-x\right)\overset{\infty }{\underset{k=0}{\sum }}\left(f_{N}\left(p_{k}\right)-f_{N}\left(l\right)\right)x^{k}\right|<\frac{ɛ}{3}. \end{array}$$

Now for 1 − *δ* < *x* < 1, it follows from (6), (7), and (8) that

(9) |(1−x)∑k=0∞(f(pk)−f(l))xk| ≤|(1−x)∑k=0∞(f(pk)−fN(pk))xk|  +|(1−x)∑k=0∞(fN(pk)−fN(l))xk|  +|(1−x)∑k=0∞(fN(l)−f(l))xk| <ɛ3+ɛ3+ɛ3=ɛ.

This completes the proof of the theorem.

In the following theorem we prove that the set of Abel continuous functions is a closed subset of the space of continuous functions.

**Theorem 15**

The set of Abel continuous functions on a subset *E* of *R* is a closed subset of the set of all continuous functions on *E*; that is, $\bar{AC(E)}=AC(E)$, where **A** **C**(*E*) is the set of all Abel continuous functions on *E* and $\bar{AC(E)}$ denotes the set of all cluster points of **A** **C**(*E*).

**Proof**

Let *f* be any element in the closure of **A** **C**(*E*). Then there exists a sequence (*f*_*n*_) of points in **A** **C**(*E*) such that lim⁡_*n*→*∞*_ *f*_*n*_ = *f*. To show that *f* is Abel continuous, take any Abel convergent sequence (*p*_*n*_) of points *E* with Abel limit *l*. Let *ɛ* > 0. Since (*f*_*n*_) is convergent to *f*, there exists a positive integer *N* such that

(10) $$\begin{array}{c} \left|f_{n}\left(t\right)-f\left(t\right)\right|<\frac{ɛ}{6} \end{array}$$

for all *t* ∈ *E* whenever *n* ≥ *N*. Write

(11) M=max⁡⁡{|f(l)−fN(l)|,|f(p1)−fN(p1)|,…,|f(pN−1)−fN(pN−1)|},

and *δ*_1_ = *ɛ*/6(*N* + 1)(*M* + 1). Then we obtain that for any *x* satisfying 1 − *δ*_1_ < *x* < 1,

(12) |(1−x)∑k=0∞(f(pk)−fN(pk))xk|≤|(1−x)∑k=0N−1(f(pk)−fN(pk))xk| +|(1−x)∑k=N∞(f(pk)−fN(pk))xk|<(1−x)MN+|(1−x)∑k=N∞(f(pk)−fN(pk))xk|<ɛ6+ɛ6=ɛ3.

As *f*_*N*_ is Abel continuous, then there exists a *δ*_2_ > 0 such that for 1 − *δ*_2_ < *x* < 1

(13) $$\begin{array}{c} \left|\left(1-x\right)\overset{\infty }{\underset{k=0}{\sum }}\left(f_{N}\left(p_{k}\right)-f_{N}\left(l\right)\right)x^{k}\right|<\frac{ɛ}{3}. \end{array}$$

Let *δ* = min⁡⁡{*δ*_1_, *δ*_2_}. Now, for 1 − *δ* < *x* < 1, we have

(14) |(1−x)∑k=0∞f(pk)xk−f(l)|≤|(1−x)∑k=0∞(f(pk)−fN(pk))xk| +|(1−x)∑k=0∞(fN(pk))−fN(l))xk| +|(1−x)∑k=0∞(fN(l)−f(l))xk|<ɛ3+ɛ3+ɛ3=ɛ.

This completes the proof of the theorem.

**Corollary 16**

The set of all Abel continuous functions on a subset *E* of *R* is a complete subspace of the space of all continuous functions on *E*.

**Proof**

The proof follows from the preceding theorem and the fact that the set of all continuous functions on *E* is complete.

**Theorem 17**

Abel continuous image of any Abel sequentially compact subset of *R* is Abel sequentially compact.

**Proof**

Although the proof follows from Theorem 7 in [2], we give a short proof for completeness. Let *f* be any Abel continuous function defined on a subset *E* of *R* and let *F* be any Abel sequentially compact subset of *E*. Take any sequence **w** = (*w*_*n*_) of point in *f*(*F*). Write *w*_*n*_ = *f*(*p*_*n*_) for each positive integer *n*. Since *E* is Abel sequentially compact, there exists an Abel convergent subsequence **r** = (*r*_*k*_) of the sequence **p**. Write Abel-lim⁡**r** = *l*. Since *f* is Abel continuous Abel-lim⁡*f*(**r**) = *f*(*l*). Thus *f*(**r**) = (*f*(*r*_*k*_)) is Abel convergent to *f*(*l*) and a subsequence of the sequence **w**. This completes the proof.

For *G* : = Abel-lim⁡, we have the following.

**Theorem 18**

If a function *f* is Abel continuous on a subset *E* of *R*, then

(15) $$\begin{array}{c} f\left({\bar{A}}^{\text{Abel}}\right)\subset {\bar{\left(f\left(A\right)\right)}}^{\text{Abel}}, \end{array}$$

for every subset *A* of *E*.

**Proof**

The proof follows from the regularity of Abel method and Theorem 8 on page 316 of [3].

**Theorem 19**

For any regular subsequential method *G*, if a subset of *R* is *G*-sequentially compact, then it is Abel sequentially compact.

**Proof**

The proof can be obtained by noticing the regularity and subsequentiality of *G* (see [2] for the detail of *G*-sequential compactness).

**Theorem 20**

A subset of  *R* is Abel sequentially compact if and only if it is bounded and closed.

**Proof**

It is clear that any bounded and closed subset of *R* is Abel sequentially compact. Suppose first that *E* is unbounded so that we can construct a sequence **p** = (*p*_*n*_) of points in *E* such that *p*_*n*_ > 2^*n*^ and *p*_*n*_ > *p*_*n*−1_ for each positive integer *n*. It is easily seen that the sequence **p** has no Abel convergent subsequence. If it is unbounded below, then similarly we construct a sequence of points in *E* which has no Abel convergent subsequence. Hence *E* is not Abel sequentially compact. Now suppose that *E* is not closed so that there exists a point *l* in $\bar{E}-E$. Then there exists a sequence of **p** = (*p*_*n*_) of points in *E* that converges to *l*. Every subsequence of **p** also converges to *l*. Since Abel method is regular, every subsequence of **p** Abel converges to *l*. Since *l* is not a member of *E*, *E* is not Abel sequentially compact. This contradiction completes the proof that Abel sequentially compactness implies boundedness and closedness.

We note that an Abel sequentially compact subset of *R* is slowly oscillating compact [15], an Abel sequentially compact subset of *R* is ward compact [24], and an Abel sequentially compact subset of *R* is *δ*-ward compact [25].

## 4. Conclusion

In this paper we introduce a concept of Abel continuity and a concept of Abel sequential compactness and present theorems related to this kind of sequential continuity, this kind of sequential compactness, continuity, statistical continuity, lacunary statistical continuity, and uniform continuity. One may expect this investigation to be a useful tool in the field of analysis in modeling various problems occurring in many areas of science, dynamical systems, computer science, information theory, and biological science. On the other hand, we suggest to introduce a concept of fuzzy Abel sequential compactness and investigate fuzzy Abel continuity for fuzzy functions (see [26] for the definitions and related concepts in fuzzy setting). However due to the change in settings, the definitions and methods of proofs will not always be the same. We also suggest to investigate a theory in dynamical systems by introducing the following concept: two dynamical systems are called Abel-conjugate if there is a one-to-one and onto function such that, *h*, and *h* ^−1^ are Abel continuous, and *h* commutes the mappings at each point. An investigation of Abel continuity and Abel compactness can be done for double sequences (see [27] for basic concepts in the double sequences case). It seems both double Abel continuity and Abel continuity coincides but it needs proving.
